# Implementing coordinated ambulatory cardiology care in southern Germany: a mixed-methods study

**DOI:** 10.1186/s12913-019-4832-4

**Published:** 2019-12-19

**Authors:** Patrick Hennrich, Regine Bölter, Michel Wensing

**Affiliations:** Department of General Practice & Health Services Research, Heidelberg University Hospital, Im Neuenheimer Feld 130.3, 69120 Heidelberg, Germany

**Keywords:** Cardiology, Managed care, Selective contract, Implementation, Mixed-methods, Ambulatory, GP-centered care

## Abstract

**Background:**

In 2009 health insurers AOK and Bosch BKK introduced the “FacharztProgramm Kardiologie” - a program for coordinated ambulatory cardiology care in the German state of Baden-Württemberg. It aims for efficient, medical guideline-oriented cardiology care to reduce avoidable hospitalizations as well as costs of care. A high number of cardiologists participate and the program has served as blueprint for programs in other medical fields. With many prerequisites and conditions involved, its implementation cannot be expected to be self-directed. Still, only little data on the actual implementation exists. We aimed to determine to what extent medical specialists and cooperating general practitioners implemented the program, which components they adapted, and which contextual factors they deemed relevant.

**Methods:**

We collected data from primary care practices of medical specialists and general practitioners within Baden-Württemberg.

Qualitative data was obtained through structured telephone interviews with participating and non-participating medical specialists as well as general practitioners cooperating with the program and general practitioners not cooperating. Interviews were analyzed through content-structuring qualitative content analyses via MAXQDA.

Quantitative data was obtained using anonymous written questionnaires completed by participating and non-participating medical specialists as well as general practitioners cooperating with the program. Analyses were performed using SPSS Statistics, mainly with regard to differences within and between groups of physicians.

**Results:**

Most components of the program regarding medical care were well implemented. However, access to medical care was not completely as intended due to high numbers of patients participating in the program and prioritization by physicians. Procedures for communication and cooperation between medical specialists and general practitioners were only partially adhered to and standardized communication was not implemented. A range of regional and practice-related contextual factors influenced implementation and outcomes.

**Conclusions:**

Implementation of this program was mixed. Contextual factors posed individual challenges to participating physicians which can’t be captured by an encompassing program. Both control mechanisms and tailoring of the program to medical care seem needed.

**Trial registration:**

Though not a clinical study, we deemed registration appropriate to ensure transparency. The study has been registered as a non-interventional observation study at the German Clinical Trials Register under ID: DRKS00013070.

## Background

While managed care first emerged in the United States of America [1], several other countries apply similar concepts. In 2009 Germany introduced “Selektivverträge” (selective contracts) based on §73c (§140a since 2015) in book five of the German Social Security Law (SGB V). The legislation allows health insurers to conclude contracts with medical specialists to provide “exceptional ambulatory medical care” for patients [2], adding to a vast range of variations in the provision of health care to foster integration within the ambulatory sector of the rather fragmented German health system [3]. Participating medical specialists are, e.g. required to strictly adhere to clinical guidelines and regulations, follow standardized methods of documentation and communication, and provide services such as faster access to medical care and a broader scope of diagnostic procedures. In return, they receive higher and more secure reimbursement compared to usual healthcare. While a substantial number of German physicians entered a selective contract in their field, insight into the actual adoption of a contract’s components in routine practice is still limited.

### Coordinated ambulatory cardiology care

In late 2009, German health insurers AOK (Allgemeine Ortskrankenkasse) Baden-Württemberg and Bosch BKK (Betriebskrankenkasse) started a program in coordinated ambulatory cardiology care together with MEDIVERBUND (independent group representing physicians’ interests) as a management organization, the Bundesverband Niedergelassener Kardiologen (nationwide association of practice-based cardiologists) and the Berufsverband niedergelassener fachärztlich tätiger Internisten (professional association of practice-based internists). Within the state of Baden-Württemberg (roughly 11 million inhabitants [4]), they initiated the “Facharztvertrag Kardiologie” (medical specialist’s contract in cardiology) as the first German selective contract, which, for patients, led to the “AOK FacharztProgramm” (AOK medical specialist’s program) being rolled-out in early 2010. Legally based on § 73c SGB V, the contract and the resulting program aim for more efficient, cost reduced and more guideline-oriented ambulatory cardiology care compared to usual ambulatory cardiology care. By definition physicians enter the contract while patients participate in the program. In the following sections we subsume the described concept of coordinated ambulatory cardiology care under the term “cardiology program”.

Considering its economic goals and focus on high quality care, the cardiology program encompasses various elements which allow the program to be categorized under the concept of managed care [5]: Selective contracting between financers and care providers ([6], p. 1), a mixed form of payment with characteristics of lump-sum and pay-for-performance reimbursements [6] and a restriction of the free choice of doctor for patients ([6], p. 7) to name a few. It contains several components both for medical specialists and patients, summarized below.

#### Access to cardiology care

The cardiology program is a medical specialist’s complement to “The AOK Family Doctor Programme” (general practitioner (GP)-centered care) in Baden-Württemberg ([6], p. 3f). Within that program, GPs are the gatekeeping primary coordinators of care [7]. Patients who wish to participate in the cardiology program need to be enrolled in GP-centered care first ([6], p. 13). By taking part in one medical specialist’s program, patients are automatically enrolled in every medical specialist’s program AOK offers and may only see participating specialists in the respective field [6]. Beforehand, they need to consult their GP who examines them and, if necessary, paves the way to specialists through referrals (except for emergencies, gynecologists, eye specialists and pediatricians) [6]. This procedure ought to avoid unnecessary consultations of medical specialists by introducing more target-oriented referrals. In return, participating patients are eligible for access-related services: The cardiology program guarantees an appointment with a medical specialist within 2 weeks or, in urgent cases if initiated by the GP, on the same day ([6], p. 8). Evening appointments until at least 8:00 p.m. need to be available for working patients once a week ([6], p. 8). Within the practice, a waiting time maximum of 30 min is promised if the patient has an appointment and no emergencies intervene ([6], p. 8).

#### Medical care

Medical specialists within the program are required to strictly adhere to cardiologic guidelines to ensure evidence-based therapy ([6], p. 9). Furthermore, they are reimbursed for a different (broader) scope of diagnostic measures than physicians in regular health care [6]. Specialists are admonished to prescribe generic medicaments geared to discount contracts between the health insurer and pharmaceutical firms ([6], p. 10). Therefore, participating practices need to equip themselves with a software feature indicating discounts through color coding. In return, physicians receive financial bonuses for prescribing these discounted pharmaceuticals [6].

#### Cooperation between specialists and GPs

Participating specialists are bound to standardized communication and cooperation with the respective GPs in GP-centered care. Diagnostic findings have to be reported to the GP within 3 days or, in case of an emergency, immediately ([6], p. 8). The specialists’ report ought to contain diagnoses, ICD-Codes (International Statistical Classification of Diseases and Related Health Problems), anamnesis, pre-medications, the patient’s status, laboratory results, instrument-based diagnostics and findings, a summed up evaluation, therapy suggestions and a range of applicable health products on offer [6]. Vice versa, GPs in GP-centered care are required to fill out a structured, accompanying letter in addition to the referral and hand it out to the patient for dissemination to the specialist. The accompanying letter needs to report (presumption) diagnoses, pre-existing medical conditions and comorbidities, information on participation of the patient in a disease-management-program, a consultation regarding health products, current medication, known drug and contrast agent intolerances, laboratory results and current instrument-based findings [6].

## Research objectives

As the cardiology program entails a high number of prerequisites and conditions, its implementation cannot be expected to be self-directed. Still, nearly 10 years after the program’s initiation, with about 70% of cardiologists and 15% of AOK-insured patients in Baden-Württemberg participating (own calculations, based on participant lists provided to us by AOK Baden-Württemberg, personal communication and [8]), there was only little insight on its implementation by medical specialists and cooperating GPs. Therefore, and in light of the efforts and costs the program causes for financers and physicians, this study aimed to answer the questions to what extent participating specialists (and GPs where applicable) actually implemented the cardiology program, which of the components mentioned they adapted, and which contextual factors influenced the implementation and outcomes from the view of participating physicians.

## Methods

As part of a larger study conducted by the University Hospitals of Jena and Heidelberg, Goethe-University Frankfurt am Main and aQua-Institute (Institute for Applied Quality Improvement and Research in Health Care) Göttingen, we performed the mixed-methods process evaluation of the cardiology program based on interviews and surveys with physicians. The Medical Research Council Guidance on process evaluations of complex interventions served as guidance for study design and procedures [9].

### Study population and sampling

For the qualitative data, we included a purposeful sample of a) medical specialists participating in the cardiology program, b) eligible, but non-participating medical specialists, c) GPs participating in GP-centered care and d) non-participating GPs. For the quantitative data, both medical specialists and GPs participating in GP-centered care were included. All practices needed to be in the state of Baden-Württemberg, as the cardiology program exists only there. As the cardiology program is predominantly directed towards adult patients, pediatricians were excluded.

Participating medical specialists and GPs in GP-centered care were identified through participant lists provided to us by AOK Baden-Württemberg. Non-participants were manually identified in the online physician’s database of the Association of Statutory Health Insurance Physicians of Baden-Württemberg [10]. Eligibility for the cardiology program is not necessarily limited to cardiologists, but can also encompass internal specialists. Thus, we searched for cardiologists as well as physicians with internal medicine as their special field who offer the disease management program for coronary heart disease, the latter at least being an indicator for cardiology-related practices. In contrast, GPs could be directly identified. Still, some non-participants might not have been covered as physicians may object to registration in the database.

To explore diverse experiences and opinions, the qualitative study aimed towards a saturation of statements in a high number of different practices instead of physicians. Otherwise there had been the risk of including several physicians from one and the same practice, limiting insight gained from the study. One hundred thirty-nine specialists’ practices participating in the cardiology program and 84 non-participating practices were identified. From 3903 GPs identified as participating in GP-centered care we drew a sample of 316 practices. Finally, we identified a total of 91 GPs’ practices not participating in GP-centered care. We contacted one physician per practice. Contact was initially established via mail, soon replaced by fax to check for general interest as this proved to be more feasible for physicians, giving them the chance to directly decline or indicate their interest in further information. Interested physicians received necessary background information and had to sign a written form of consent before an appointment for the interview was made. Confidentiality was assured through pseudonyms to prevent bias resulting from socially desirable answers.

The quantitative study focused on individual physicians regardless of the respective practices. In updated lists and searches 212 specialists participating in the cardiology program, 99 non-participating specialists and 3599 participating GPs were identified. Both groups of specialists were contacted entirely, a total random sample of 400 GPs was drawn. Physicians received a postal quantitative questionnaire with an accompanying letter and a self-addressed stamped envelope. Completing and returning the questionnaire was interpreted as consent, the questionnaire was anonymous to ensure confidentiality and prevent bias resulting from socially desirable answers.

### Data collection and measures

From 09/2017 to 07/2018 qualitative data were collected through structured, guideline-based telephone interviews preceded by a short, written questionnaire (see Additional file 1) on sociodemographic data for sample description. Guidelines were constructed from components of the cardiology program and insights gained during pre-tests. Depending on the group interviewed, guidelines spanned up to six different topics: a) the physician’s motivation (not) to participate in (or, for GPs, cooperate with) the cardiology program as well as their expectations, b) their day-to-day tasks and changes they noticed since introduction of the program, c) cooperation with other care providers, d) questions on how they organized their practices, e) factors affecting implementation of the program and f) the program’s concomitants (see Additional file 2 for all questions related to implementation of the cardiology program). Interviews were intended to last 30–40 min. The guideline was pilot tested in interviews with 3 different physicians.

From 10/2018 to 01/2019 quantitative data were collected through the anonymous questionnaires already mentioned (see Additional file 1). Questions were related to components of the cardiology program and enhanced by insights gained from the qualitative data. The questionnaires were constructed by the researchers, using semantic differential scales, multiple choice questions, dichotomous questions, open ended questions and spanned up to four topics: a) General information on the physicians and their practices (17–22 questions), b) participation in and implementation of the cardiology program (9–16 questions), c) healthcare within the respective practice (6–12 questions) and d) questions on cooperation with other care providers (9–12 questions). All questionnaires were previously pilot tested with physicians and slightly adapted.

### Data-analysis

The transcribed qualitative interviews were analyzed using the qualitative data analysis software MAXQDA 2018, following the scheme of a content-structuring qualitative content analysis as described by Kuckartz [11], in light of the research objectives: Texts were manually scanned by the researchers for important and possibly relevant passages, interesting aspects were noted through memos created with the software. Then main categories were deductively derived from the research questions, interview guidelines and inductively emerging from topics mentioned by interviewees. A first code system was created from the main categories using the software. All main categories were applied to parts of the empirical material and concerted between researchers to ensure quality and precision in definitions (see also [12]). The concerted main categories were applied to the whole material and further differentiated. Corresponding sub-categories were created inductively on the basis of statements within the interviews. This resulted in the final code system which was once again concerted between researchers, applied to all transcripts via the software and interpreted subsequently with respect to the main topics of the study and in consideration of frequencies of mentions.

The quantitative data obtained was tested for normal distribution using the Kolmogorov-Smirnov-Test in SPSS 24. Afterwards it was analyzed descriptively with a focus on mean values and standard deviations (sd) (median values and corresponding interquartile range (IQR) where data was not normally distributed) as well as frequencies. To test for significant differences between participants and non-participants, we used Pearson’s Chi or, when the assumed cell count of at least 5 per cell was not reached, Fisher’s exact test. Metric data largely proved to be not normally distributed, hence comparisons were made using a two-tailed non-parametric Mann-Whitney-U-Test. Within groups we performed a two-tailed Wilcoxon-test. Significant differences relevant to the research objectives are marked in each table. For mean and median values, the corresponding n is given in square brackets where data was missing. Frequencies are given in absolute numbers with valid percentages respectively.

## Results

Qualitative interview study: Of the 139 contacted specialists participating in the cardiology program, 23 (16.5%) agreed to participate in the qualitative interview study. Out of the 23 specialists, 2 repeatedly could not be reached to make an appointment, 21 were interviewed. Of the 84 non-participating specialists, 1 turned out to be deceased while 11 (13.3%) participated in the qualitative interview study. Of the 316 contacted GPs in GP-centered care, 1 turned out to be deceased while 19 (6.0%) participated in the study and were interviewed. Of the 91 non-participating GPs, 7 (7.7%) participated in the study and were interviewed. Table 1 shows sociodemographic characteristics of respondents in the qualitative interview study. Interview length varied from 19 to 65 min with a mean of roughly 35 min.

**Table 1:** Sociodemographic characteristics of interviewed physicians in the qualitative interview study (medical specialists and GPs, each participating or not in the cardiology program and GP-centered care respectively)

Characteristic	Specialists (participating) *n* = 21	Specialists (non-participating) *n* = 11	GPs (participating) *n* = 18^a^	GPs (non-participating) *n* = 8^a^
Sex (n (%))				
male	17 (81.0)	9 (81.8)	14 (77.8)	5 (62.5)
female	4 (19.0)	2 (18.2)	4 (22.2)	3 (37.5)
Age (median (IQR))	57 (53–60)	49 (41–54)	58 (50–69)	60 (48–65)
Years of professional experience (median (IQR))	29.0 (23.5–32.0)	20.0 (13.0–25.0)	29.5 (20.0–38.5)	29.5 (21.3–36.8)
Practice-based since the year (median (IQR))	2001 (1995–2005)	2014 (2005–2017)	2002 (1993–2006)	1998 (1991–2013)
Vocational training (specialists) (n (%))				
Specialist for internal medicine …	19 (90.5)	5 (45.5)		
without focus	3 (14.3)	0 (0.0)		
with focus on cardiology	16 (76.2)	5 (45.5)		
Specialist for internal medicine and cardiology	3 (14.3)	5 (45.5)		
Other	3 (14.3)	2 (18.2)		
Vocational training (GPs) (n (%))				
Specialist for general practice			9 (50.0)	4 (50.0)
Internist working as a GP			9 (50.0)	4 (50.0)
Other			3 (16.7)	0 (0.0)
Practice location (n (%))				
city core	11 (55.0)	7 (70.0)	3 (17.6)	5 (62.5)
urban hinterland (∼20 km/12.5 mi)	4 (20.0)	2 (20.0)	6 (35.3)	1 (12.5)
rural area	5 (25.0)	1 (10.0)	8 (47.1)	2 (25.0)
Type of practice (n (%))				
individual practice	6 (30.0)	7 (70.0)	8 (44.4)	6 (75.0)
shared practice	2 (10.0)	1 (10.0)	1 (5.6)	1 (12.5)
group practice	11 (55.0)	2 (20.0)	9 (50.0)	1 (12.5)
ambulatory healthcare center	1 (5.0)	0 (0.0)	0 (0.0)	0 (0.0)
Individual patients per quarter (n (%))				
< 500	0 (0.0)	1 (9.1)	0 (0.0)	2 (25.0)
500–1000	7 (33.3)	5 (45.5)	4 (22.2)	3 (37.5)
1001–1500	6 (28.6)	4 (36.4)	7 (38.9)	2 (25.0)
> 1500	8 (38.1)	1 (9.1)	7 (38.9)	1 (12.5)
Number of full-time positions (doctors) (mean (sd)) [n]	2.9 (3.4) [20]	1.7 (1.0)	1.9 (1.1)	1.4 (0.5)
Number of full-time positions (physician's assistants) (median (IQR)) [n]	4.0 (3.3–6.5)	3.30 (2.0–5.3) [9]	3.0 (2.0–4.5)	1.6 (1.0–2.4)
Percentage of (AOK)-patients participating in the cardiology program (median (IQR)) [n]	40.0 (30.0–60.0) [19]		25.0 (10.0–60.0) [11]	
Participation in the cardiology program since the year (mean (sd)) [n]	2011 (1.2) [19]			

^a^One GP entered GP-centered care just in the timespan between the short questionnaire and the interview, so here 18 GPs are listed as participating instead of 19

Quantitative study: Of the 212 contacted specialists participating in the cardiology program, one mailing was returned to sender. Of the remaining 211 specialists, 75 (35.5%) returned their questionnaire. Of the 99 non-participating specialists, 21 (21.2%) returned their questionnaire. From the total sample of 400 GPs participating in the GP program, one mailing was returned by the corresponding practice because the physician retired shortly before our mailing arrived. Of the remaining 399 GPs, one returned questionnaire was left completely blank and therefore unusable while 73 (18.3%) were completed. Table 2 shows sociodemographic characteristics of respondents in the quantitative study.

**Table 2:** Sociodemographic characteristics of physicians in the quantitative study (medical specialists participating or not in the cardiology program and GPs participating in GP-centered care)

Characteristic	Specialists (participating) *n* = 75	Specialists (non-participating) *n* = 21	GPs (participating) *n* = 73
Sex (n (%))			
male	60 (80.0)	18 (85.7)	52 (71.2)
female	15 (20.0)	3 (14.3)	21 (28.8)
Age (median (IQR)) [n]	56 (51–60)	54 (45–57)	59 (54–65)
Years of professional experience (median (IQR))	28.0 (23.0–32.0)	23.0 (18.5–29.7)	30.0 (25.0–37.0)
Practice-based since the year (mean (sd))	2003 (8.2)	2009 (8.5)	1997 (10.6)
Vocational training of specialists (n (%))			
Specialist for internal medicine …	62 (82.7)	16 (76.2)	
without focus	9 (13.0)	1 (4.8)	
with focus on cardiology	47 (68.1)	15 (71.4)	
Specialist for internal medicine and cardiology	22 (29.3)	6 (28.6)	
Other	6 (8.0)	2 (9.5)	
Vocational training of GPs (n (%))			
Specialist for general practice			57 (78.1)
Internist working as a GP			16 (21.9)
Other			4 (5.5)
Practice location (n (%))			
city core	53 (72.6)	12 (57.1)	23 (32.9)
urban hinterland (∼20 km/12.5 mi)	8 (11.0)	6 (28.6)	27 (38.6)
rural area	12 (16.4)	3 (14.3)	20 (28.6)
Type of practice (n (%))			
individual practice	17 (23.6)	7 (33.3)	36 (49.3)
shared practice	14 (19.4)	2 (9.5)	8 (11.0)
group practice	37 (51.4)	10 (47.6)	26 (35.6)
ambulatory healthcare center	4 (5.6)	2 (9.5)	3 (4.1)
Individual patients per quarter (n (%))			
< 500	2 (2.7)	3 (14.3)	0 (0.0)
500–1000	34 (45.9)	10 (47.6)	14 (19.2)
1001–1500	21 (28.4)	3 (14.3)	36 (49.3)
> 1500	17 (23.0)	5 (23.8)	23 (31.5)
Number of full-time positions (physicians) (mean (sd)) [n]	3.0 (2.7) [66]	2.3 (2.1) [20]	2.1 (1.4) [69]
Number of full-time positions (physician's assistants) (n (%))			
0 up to 3	17 (22.7)	4 (20.0)	37 (52.1)
More than 3, up to 6	27 (36.0)	10 (50.0)	24 (33.8)
More than 6, up to 10	18 (24.0)	3 (15.0)	7 (9.9)
More than 10	13 (17.3)	3 (15.0)	3 (4.2)
Percentage of (AOK)-patients participating in the cardiology program (mean (sd)) [n]	19.4 (11.8)		33.0 (25.1) [62]
Participation in the cardiology program since the year (mean (sd)) [n]	2012 (2.6) [60]		

### Implementation of the cardiology program

#### Access to cardiology care

When it comes to accessing care within the cardiology program, the GP is planned to be the primary gatekeeper to ambulatory medical specialist care within the program and waiting times for patients are supposed to be limited. The actual implementation proved to be highly diverse as Table 3 shows.

**Table 3:** Medical specialists’ (participating or not in the cardiology program) perceptions of aspects related to accessing ambulatory cardiology care

Question	Specialists (participating) (*n* = 75)	Specialists (non-participating) (*n* = 21)
How long do patients participating in the cardiology program need to wait for an appointment? (n (%))*		
Up to two weeks	27 (37.0)	
More than two weeks, up to a month	30 (41.1)	
More than a month, up to three months	12 (16.4)	
More than three months	4 (5.5)	
How long do patients in regular healthcare need to wait for an appointment (n (%))		
Up to two weeks	4 (5.5)	1 (4.8)
More than two weeks, up to a month	6 (8.2)	3 (14.3)
More than a month, up to three months	20 (27.4)	5 (23.8)
More than three months	43 (58.9)	12 (57.1)
Is it possible to make an urgent/emergency appointment with you on the same day? (n (%))		
Yes	67 (91.8)	19 (90.5)
No	6 (8.2)	2 (9.5)
How much time does an appointed patient normally spend in your waiting room? (n (%))		
Up to 30 min	49 (68.1)	18 (85.7)
More than 30 min, up to 60 min	21 (29.2)	3 (14.3)
More than 60 min, up to 90 min	2 (2.8)	0 (0.0)
More than 90 min	0 (0.0)	0 (0.0)
Do you offer consultation hours until at least 8 p.m. once or several days a week? (n (%)) †		
Yes, regularly	30 (40.0)	2 (9.5)
Yes, but only in exceptional cases	15 (20.0)	1 (4.8)
No	30 (40.0)	18 (85.7)

* Significant difference to waiting time in regular health care within the group of participating medical specialists in asymptotic Wilcoxon-test (*p* < .001)

† Significant difference between participating and non-participating medical specialists in Fisher’s exact test (*p* < .01)

The expected appointment within 2 weeks was only partially adhered to, while most physicians stated that waiting time for an appointment can be up to 1 month or longer. Qualitative results indicated this might be based on the high numbers of patients participating in the cardiology program or prioritization issues (statements are translated from German into English and annotated with “ID-[#]” to differentiate between physicians; see Additional file 3 for additional statements and a list of qualitative analysis themes/categories relevant to the research questions).

ID-1: “There are so many participants, I don’t have any space left, I need to postpone all of them and then I repeatedly wonder: ‘Oh my god, this critically ill person waited for three months for an appointment. According to the specification in the cardiology program he should have been seen much earlier.’ But I don’t have any appointments left, all of them are already assigned – months in advance. [ … ]”

ID-2: “And the right to get an appointment within 14 days, we handle this by medical indication and urgency. And if somebody, which happens a lot of times, wants or needs to, for example, have their bypass checked [ … ] and misses the appointment or forgot that they always did this in the summer but now want it before their holiday in August by all means and call us on July 15^th^ thinking they get an appointment within two weeks because they participate in the cardiology program – that’s not going to work. And it wouldn’t work for a private patient either.”

Waiting time differences in regular healthcare proved to be insignificant between participating and non-participating physicians. However, within the group of participants, self-reported waiting time for an appointment differed significantly between the cardiology program and regular care (asymptotic Wilcoxon-test: z = − 6.78, *p* = .000, *n* = 73; r = .79).

Extended consultation hours until 8:00 p.m. once a week were not regularly offered by most participating specialists. Qualitative results indicated this might have been connected to misconceptions of the required opening hours, little demand and substitutions of late appointments with early appointments.

ID-3: “So, we have relatively late consultation hours until … 6:30 p.m. [ … ] In the beginning, we had consultation hours until 8:00 p.m. and after one or two years there was so little demand that we said: ‘No.’”

ID-1: “We have, appropriate to the requirements, late consultation hours until 7:00 p.m. now. We didn’t have this before.”

ID-4: “Yes, we start at 7:30 a.m., so that’s rather in the early morning.”

However, there were significant differences compared to non-participants regarding extended consultation hours, as non-participants were less likely to offer them (Fisher’s exact test(2) = 13.331, *p* = .001, *n* = 96; Cramer-V = .378, *p* = .001).

#### Medical care

The uptake of recommended care-related aspects within the cardiology program seemed to be implemented well. As Table 4 shows, self-reported adherence to cardiologic guidelines in therapy within the cardiology program was high on a scale from 1 (no adherence to cardiologic guidelines at all) to 6 (exclusive adherence to guidelines). Compared to non-participating specialists, there was no statistically significant difference. Furthermore, most participating specialists stated that they prescribed generic/discounted medications as often as or more often than in regular care. High guideline-adherence and widespread prescription of generic medications were also backed by the majority of participating physicians’ statements in the qualitative interviews (see Additional file 3).

**Table 4:** Physicians' (medical specialists participating or not in the cardiology program and GPs participating in GP-centered care) perceptions of medical care and cooperation

Question	Specialists (participating) (*n* = 75)	Specialists (non-participating) (*n* = 21)	GPs (participating) (*n* = 73)
In your day-to-day work, how much do you adhere to cardiologic guidelines? (mean (sd)) [n]	5.1 (0.7) [73]	5.1 (0.3) [20]	
Because of the color coding in your practice’s software you prescribe discounted medications to participating patients … (n (%))			
Less frequently than in regular care	0 (0.00)		
As frequently as/similar to regular care	37 (51.4)		
More frequently than in regular care	32 (44.4)		
I don’t know about the color coding	3 (4.2)		
When are your reports of diagnostic findings sent out to the GP? (n (%)) *			
On the same day	33 (45.2)	3 (14.3)	
Within 3 days	26 (35.6)	14 (66.7)	
Within 5 days	8 (11.0)	3 (14.3)	
Within 6 days or more	6 (8.2)	1 (4.8)	
When it comes to patients participating in the cardiology program, how often do you receive the GPs' structured “accompanying letter to the specialist” with relevant laboratory results and (presumption) diagnoses? (mean (sd)) [n]	2.0 (1.3) [73]		
When it comes to referrals of patients participating in the cardiology program, how often do you use the structured “accompanying letter to the specialist” with relevant laboratory results and (presumption) diagnoses? (mean (sd))			2.0 (1.8) [70]
Which of the following information are included in your reports to the GP? (n (%))			
Diagnoses	74 (100.0)	21 (100.0)	
ICD-Codes **	34 (45.9)	1 (4.8)	
Anamnesis	73 (98.6)	21 (100.0)	
Pre-medications	58 (78.4)	17 (81.0)	
Status	56 (75.7)	14 (66.7)	
Laboratory results	50 (67.7)	11 (52.4)	
Instrument-based diagnostics and findings	74 (100.0)	21 (100.0)	
Summed up evaluation	74 (100.0)	21 (100.0)	
Therapy suggestions	73 (98.6)	21 (100.0)	
Health products	6 (8.0)	3 (14.3)	

* Significant difference between participating and non-participating medical specialists in Fisher’s exact test (*p* < .05)

** Significant difference between participating and non-participating medical specialists in Pearson’s Chi (*p* < .01)

#### Cooperation between specialists and GPs

As Table 4 indicates, communication speed proved to be largely as intended for participating physicians and differed significantly from non-participants (Fisher’s exact test = 8.307, *p* = .030, *n* = 94; Cramer-V = .295, *p* = .042). We asked specialists how often they received the accompanying letter GPs are ought to send with every referral of patients within the cardiology program on a scale from 1 (it had never been received) to 6 (it is always being received). Of all specialists, 76.8% indicated a value between 1 and 3 while 15.0% indicated a value between 4 and 6 on the scale. An additional 8.2% claimed they did not know about the existence of the document at all. Vice-versa, we asked GPs how often they sent the accompanying letter on a scale from 1 (it is never sent) to 6 (it is always being sent). Of all GPs, 60.0% indicated a value between 1 and 3 while 22.9% indicated a value between 4 and 6 on the scale. Additionally, 17.1% claimed they did not know about the existence of the document at all. This heterogeneous range of structured communication and contact in general was also reported by physicians in the qualitative study (see Additional file 3).

The majority of designated contents of the specialists’ reports to the GPs were reported in most cases according to medical specialists. However, appropriate ICD-codes and an overview of suitable health products on offer were seldom given. Still, with ICD-codes there was significant difference in reporting between participating and non-participating specialists (Chi^2^(1) = 11.924, *p* = .001, *n* = 95; Phi = .354, *p* = .001).

### Contextual factors

Implementation and outcomes of the cardiology program were apparently influenced by several contextual factors. Additional statements on these aspects are shown in Additional file 3.

#### Region

Qualitative analyses showed that implementation of the cardiology program might have been influenced by the region in which a practice is based. Specialists named local presence of the initiating health insurer as but one aspect determining how the program translates into practice. Additionally, distribution of GP-centered care was important: If only a minority of GPs participated in GP-centered care, cooperation may not have worked as intended.

ID-5: “[ … ] the only issue I hear from colleagues: If AOK is not or little represented or no GPs participate in the program within the respective region. In the region of South Baden, regions where only few GPs participate [*in GP-centered care*], colleagues state that participating [*in the cardiology program*] is difficult.”

Furthermore, there were professional political aspects: Several participating and non-participating physicians reported reservations concerning the organizations behind the cardiology program within certain regions of Baden-Württemberg.

ID-6: “[ … ] like I said, in some regions there is a political orientation which is rather hostile towards the management corporation. It’s fewer of an issue with the contract itself than with the management corporation. This might have been an influence on several colleagues in said regions, obviously, to … rather not to participate.”

#### Practice-based factors

Practices participating in the cardiology program differed from each other when it came to infrastructure, personnel and composition of special fields. Implementation therefore posed highly diverse challenges to them.

##### Staff

While most medical specialists deemed their practices well-equipped regarding staff, some stated that their staff is unable to cope with the additional workload, either for pure quantitative reasons or because of a lack of necessary skills.

ID-2: “We have two physician assistants who are well versed in it [*the cardiology program*]. The others say: ‘Hands off!’ if there is a participating AOK-patient. Partially, they don’t even want to deal with it which leads to some things being forgotten”

##### Administrative infrastructure

Another factor was each practice’s administrative infrastructure regarding the practice’s organization and software. The cardiology program forces physicians to execute several day-to-day processes twice: One time for their “regular” patients and one time for participants in the program.

ID-2: “These patients aren’t very popular – not the patients as such, but when it comes to accounting they are not very popular. And it … I don’t know about the situation in other practices, at our place thoughts are more like: ‘Oh dear, he’s participating’.”

Additionally, not all practices were only active in the field of cardiology but, especially in group practices or ambulatory healthcare centers, combined several disciplines which were sometimes incompatible to the regulations set by the cardiology program or requested even more repetitions of certain processes.

ID-7: “I have patients participating in the cardiology program. That’s one accounting. Then I have patients who are members of a statutory health fund – these are different numbers. Then there are my wife’s patients who are members of a statutory health fund. Now we have three accountings. Then there are the ones [*only*] participating in GP-centered care, four accountings. And then there are the ones in private healthcare as number five. So that’s the difficulty, a physician assistant can’t overlook it well: Which number has to be recorded where? This means that I need to invest a lot of time in the evening to check accountings myself to prevent mistakes. In the beginning there were a lot of them, meanwhile I learned it to some degree. Then there are penalties if you do it wrong. That’s not nice, of course. But if you have five different accountings, possibilities, five different versions, then a lot of mistakes happen and this doesn’t make it [*accounting*] easier. [ … ] I think if I was a sole specialist, it [*handling the cardiology program*] would be much easier of course. I mean being alone in a specialist’s practice and not one practice covering several special fields. That’s the problem. This is a group practice with several special fields.”

## Discussion

The uptake of the key components of the cardiology program was mixed. While recommended medical care seemed well implemented, access to cardiology care as well as information exchange between medical specialists and GPs showed room for improvement. Several context factors were perceived to influence the uptake of program components, including features of practice organization and the regional number of GPs in GP-centered care.

Access to medical care in the cardiology program did not always meet expectations: Appointments within 2 weeks were often impossible due to high numbers of participating patients and the need to structure appointments by urgency. High participation rates within a practice might undermine the concept of fast appointments, as every participant is eligible. This makes the right to an appointment within 2 weeks the rule and not the exception. One must not be surprised that specialists prioritize with regard to urgency then – in fact, this can be seen as their duty as otherwise patients might not get proper treatment in time.

Extended consultation hours were merely implemented, either because of a misunderstanding of required opening times, a substitution for consultation hours in the early morning or little demand. It is questionable if late appointments need to be mandatory when demand is missing. Internationally, a UK study showed that, at least for GPs, even extended consultation hours outside of the weekend have no significant effect on patients’ demands compared to practices with regular consultation hours [13]. Flexible regulations aimed at actual demand seem more appropriate here.

Nearly all specialists in the study reported high adherence to clinical guidelines – regardless of participation in the cardiology program. Even though implementation of guidelines can be difficult [14], high perceived adherence comes as no surprise: Guidelines are omnipresent in cardiology – in the past 3 years, the European Society of Cardiology alone published four to five new guidelines per year [15]. Though not legally binding in Germany, under certain circumstances guideline-adherence works as a defensive tool against accusations of malpractice [16, 17]. Strong differences between participating and non-participating physicians therefore would be rather surprising.

Efficient care is heavily affected by cooperation and coordination between involved practitioners [18–21]. This is still problematic within the cardiology program: while information exchange seems to be fast, standardization in the form of a structured accompanying letter has not been implemented successfully. Information still varies in content and extent, similar to past studies on communication between physicians [22–24]. What meets the eye is that regulations concerning cooperation and communication can, compared to accounting data or prescriptions, not be screened by the health insurers in charge. This may hinder sanctioning, making deviant behavior easier and less fraught with risk than in a completely checkable situation. On the other hand, such a “reimagined” implementation of the contract’s regulations by physicians also matches reinvention processes described in past research on the diffusion of innovations: Innovations (here: the cardiology program with its regulations) may be changed by adopters (here: physicians) after adoption according to their needs, e.g. because of a mismatch between the innovation and the actual situation adopters are in [25]. Probably the standardization measures for communication within the cardiology program (the same goes for regulations on extended consultation hours we discussed above) did simply not suit cardiology care reality sufficiently and were therefore less adhered to by physicians than other aspects of the program. In the long run, the situation may become similar to managed care programs in the United States during the early 2000s: Major problems emerged regarding strict regulations of programs. Eventually, this caused managed care organizations to include less and less restrictive specifications and more freedom of action in their programs [26, 27]. The cardiology program aims for standardization to create reliable, complete reports between physicians and is not meant to simply keep up lines of action that it is supposed to change. However, reality of care might force a transition in the program’s strictness comparable to the situation in the US described above.

Finally, we showed that adoption of the program depended on contextual factors – the region a practice is based in influenced implementation as well as practice-based factors such as staff and administrative infrastructure. Similar contextual factors were observed in other countries: In the heyday of managed care in the USA during the 1990s, physicians already reported difficulties regarding administrative efforts compared to regular health care [28]. The role of contextual factors is important regarding limitations of the contracts typically underlying managed care programs (here: the cardiology contract): Encompassing contracts are always written out broadly, still there needs to be a certain scope allowing for different contexts.

From a more methodological perspective, the specific themes and results of our analyses might probably function as an onset for an evaluation framework specifically tailored to selective contracts in the German context. This, however, demands further research to see if our themes and results can be found in other contracts and programs of this kind. Especially the fact that selective contracts are often tailored to single states within Germany and not to the country as a whole does not yet allow for definite assumptions on the transferability of themes nationwide.

### Limitations

Being part of the first evaluation of a German selective contract for medical specialists, this study has a pioneering role. The mixed-methods approach was advantageous, as some questions the quantitative results brought up could be answered with qualitative data and vice versa. Still, there are limitations: There is no ex ante data on behavior and communication of specialists before they participated in the program, leaving room for systematic differences between participants and non-participants. For identification of non-participants, we had to rely on a database where physicians are not bound to be registered. There might be non-participants we weren’t able to identify because they (systematically) do not enlist in such databases. This leaves room for a biased sample. Last, physicians had to report on their own behavior, possibly leading to socially desirable answers regardless of ensured confidentiality.

A question that our data is unable to answer: Did specialists in the cardiology program behave differently prior to participation or did they fulfill most criteria anyway? A contract might not be able to change physicians’ behavior in such a strong manner that significant differences to those not entering the contract emerge. Participation in the cardiology program might rather be a sign of quality, signalizing standards already present instead of improving the status quo. Future research should therefore collect ex ante data to allow before-after-comparisons and direct statements on effects of such programs within the group of participating physicians. Furthermore, it would be interesting to conduct another study in 5 to 10 years to see if the future of the cardiology program will eventually be similar to other managed care programs such as those in the USA, as some of our findings already resemble the onset of trends observed overseas.

## Conclusions

Being the first German medical specialist-related selective contract, the cardiology contract still poses as an example for other selective contracts and resulting programs. Providing a first insight into the implementation of a program based on § 73c SGB V and showing accompanying challenges, our study delivers knowledge for the program’s improvement and the development of future selective contracts.

Eight years after the cardiology program came into full effect, several components seemed to be implemented well while others were still lacking complete translation to medical care. Possible reasons we discussed were a lack of controllability of behavior, an increase in participating patients and infeasibility of regulations due to contextual factors. Our findings can pave the way for reforms of the existing program and deliver useful insights for future selective contracts. In our case, policymakers may reevaluate the cardiology program, especially regarding the feasibility of measures on aspects such as cooperation between physicians, as deviant behavior is already present. This might hint at a need for a more specific tailoring to medical care reality and the individual situation of practices. For similar (future) programs, room for these individual needs of participating physicians seems needed, always in light of a thorough consideration of medical care reality before a program starts. Furthermore, more extensive support for the implementation might be adequate to avoid implementation discrepancies due to misconceptions, like we observed them regarding consultation hours. Like our process evaluation, lots of health services research bears a rather practical relevance, as health systems around the world are highly heterogeneous and cannot be compared easily. Still, some of our results could be linked to international concepts of managed care and parallels emerged, showing possible perspectives for managed care programs in general and the cardiology program in particular.

## Supplementary information

**Additional file 1.** Questionnaires.

**Additional file 2.** Questions from the qualitative interview guides on implementation of the cardiology program.

**Additional file 3.** Themes/categories and representative quotes from the qualitative data analysis relevant to the implementation of the cardiology program.

## Data Availability

The datasets used and analyzed during the current study are available from the corresponding author on reasonable request.

## Abbreviations

AOK: Allgemeine Ortskrankenkasse [German health insurance company]
aQua-Institute: Institute for Applied Quality Improvement and Research in Health Care
Bosch BKK: Bosch Betriebskrankenkasse [German health insurance company]
DMP: Disease management program
EFA: Entlastungsassistentin in der Facharztpraxis [type of physician’s assistant]
GP: General practitioner
ICD: International Statistical Classification of Diseases and Related Health Problems
IQR: Interquartile range
MAXQDA: Qualitative data analysis software
n: Sample size
p: Probability value
r: Correlation coefficient
sd: Standard deviation
SGB V: Fünftes Sozialgesetzbuch [book five of the German Social Security Law]
z: Z-score
