# Supplementary material for: Implementing coordinated ambulatory cardiology care in southern Germany: a mixed-methods study
Source: BMC Health Serv Res. 2019 Dec 19;19:976. doi: 10.1186/s12913-019-4832-4 (PMC6921457; doi:10.1186/s12913-019-4832-4)
Supplement: Supplementary file 1 — Additional file 1. Questionnaires. [file 12913_2019_4832_MOESM1_ESM.docx]

# Additional file 1: Questionnaires*

## 1. Short questionnaire (preceding qualitative telephone interviews) for cardiologists participating in the cardiology program

| **Year of birth:** | 19 \|___\|___\| (Please insert year) | | | |
| --- | --- | --- | --- | --- |
| **Sex:** | female | male | | |
| **How many years of professional experience do you have?** | ca. \|___\|___\| years (including further education) | | | |
| **In which year did you take up residence?** | \|___\|___\|___\|___\| (Please insert year) | | | |
| **What is your field of expertise:** | Internal medicine: | without focus  with focus on cardiology | | |
|  | Internal medicine and cardiology  Other: _______________________________ | | | |
| **Which additional qualifications do you have?** | 1. ________________________________________  2. ________________________________________  3. ________________________________________ | | | |
| **What are the main fields of your practice?** | 1. ________________________________________  2. ________________________________________  3. ________________________________________ | | | |
| **Where is your practice located?** | City center  Urban hinterland (ca. 20 km) | | | Rural area |
| **In which type of practice do you work?** | Individual practice  Shared practice | | | Ambulatory healthcare center  Group practice |
| **How many individual patients do you see per quarter?** | < 500  1001-1500 | | | 500-1000  > 1500 |
| **What is the total full time equivalent of physicians in your practice?** | \|___\|___\|,\|___\| full time physicians | | | |
| **What is the total full time equivalent of physicians’ assistants in your practice?** | \|___\|___\|,\|___\| full time physicians’ assistants | | | |
| **Do you employ an EFA in cardiology [type of assistant for medical specialist’s practices]** | No  Yes, \|___\|___\| EFA | | | |
| **How do you handle documentation in your practice?** | completely digital  completely paper- based  partly digital, partly paper-based | | | |
| **Do you participate in one or more disease management programs?** | No  Yes, heart failure module | | Yes, coronary heart disease  Other | |
| **How many of your patients suffering from coronary heart disease participate in the disease management program for coronary heart disease?** | ca. \|___\|___\|___\| % of patients with coronary heart disease  not applicable | | | |
| **How many of your patients suffering from heart failure participate in the heart failure module?** | ca. \|___\|___\|___\| % of patients with heart failure  not applicable | | | |
| **How many urgent cardiologic emergencies do you have per month that require further diagnostics or a referral to the hospital?** | ca. \|___\|___\|___\| urgent emergencies per month | | | |

| **Since when do you participate in the cardiology program?** | 20 \|___\|___\| (Please enter year) | | | | | | |
| --- | --- | --- | --- | --- | --- | --- | --- |
| **How complex was integration of the cardiology program in your day-to-day business?** |  |  |  |  | |  |  |
|  | Not complex  at all | |  | | | Very  complex | |
| **How many of your (AOK-) patients participate in the cardiology program?** | ca. \|___\|___\|___\| % | | | | | | |
| **Which people are primarily responsible for implementation of the cardiology program in your practice?** | Myself  Physician’s assistants  Other: _________ | | | | Other physicians  EFA | | |
| **How many times a year do you participate in cardiologically relevant general practitioner’s quality circles on pharmaceutical therapy?** | ca. \|___\|___\| times a year | | | | | | |
| **How often do you participate in advanced education with a focus on cardiology?** | ca. \|___\|___\| times a year | | | | | | |
| **Do you use a cardiology contract-related software for prescriptions of pharmaceuticals?** | Yes  No | | | | | | |
| **Do you hand out further (evidence-based) information to your patients on managing their illness?** | No  Yes, e.g. from the following sources: __________________________________________________________________________________________________________________ | | | | | | |
| **On a regular base, do you counsel your patients on health and prevention offers?** | Yes  No | | | | | | |
| **On a regular base, are you in touch with an AOK prevention consultant?** | Yes  No | | | | | | |
| **Do you use a digital quality management system?** | Yes, ______________ (please specify)  No | | | | | | |
| **Please rate your content with the cardiology program through school grades** (1= „very good“, 6 = „insufficient“) | Grade: _______________ | | | | | | |

## 2. Short questionnaire (preceding qualitative telephone interviews) for cardiologists not participating in the cardiology program

| **Year of birth:** | 19 \|___\|___\| (Please insert year) | | | | |
| --- | --- | --- | --- | --- | --- |
| **Sex:** | female | male | | | |
| **How many years of professional experience do you have?** | ca. \|___\|___\| years (including further education) | | | | |
| **In which year did you take up residence?** | \|___\|___\|___\|___\| (Please insert year) | | | | |
| **What is your field of expertise:** | Internal medicine: | without focus  with focus on cardiology | | | |
|  | Internal medicine and cardiology  Other: _______________________________ | | | | |
| **Which additional qualifications do you have?** | 1. ________________________________________  2. ________________________________________  3. ________________________________________ | | | | |
| **What are the main fields of your practice?** | 1. ________________________________________  2. ________________________________________  3. ________________________________________ | | | | |
| **Where is your practice located?** | City center  Urban hinterland (ca. 20 km) | | | Rural area | |
| **In which type of practice do you work?** | Individual practice  Shared practice | | | Ambulatory healthcare center  Group practice | |
| **How many individual patients do you see per quarter?** | < 500  1001-1500 | | | 500-1000  > 1500 | |
| **What is the total full time equivalent of physicians in your practice?** | \|___\|___\|,\|___\| full time physicians | | | | |
| **What is the total full time equivalent of physicians’ assistants in your practice?** | \|___\|___\|,\|___\| full time physicians’ assistants | | | | |
| **Do you employ an EFA in cardiology [type of assistant for medical specialist’s practices]** | No  Yes, \|___\|___\| EFA | | |  | |
| **How do you handle documentation in your practice?** | completely digital  completely paper- based  partly digital, partly paper-based | | | | |
| **Do you participate in one or more disease management programs?** | No  Yes, heart failure module | | Yes, coronary heart disease  Other | | |
| **How many of your patients suffering from coronary heart disease participate in the disease management program for coronary heart disease?** | ca. \|___\|___\|___\| % of patients with coronary heart disease  not applicable | | | |  |
| **How many of your patients suffering from heart failure participate in the heart failure module?** | ca. \|___\|___\|___\| % of patients with heart failure  not applicable | | | | |
| **How many urgent cardiologic emergencies do you have per month that require further diagnostics or a referral to the hospital?** | ca. \|___\|___\|___\| urgent emergencies per month | | | | |
| **How many times a year do you participate in cardiologically relevant general practitioner’s quality circles on pharmaceutical therapy?** | ca. \|___\|___\| times a year | | | | |
| **How often do you participate in advanced education with a focus on cardiology?** | ca. \|___\|___\| times a year | | | | |
| **Do you hand out further (evidence-based) information to your patients on managing their illness?** | No  Yes, e.g. from the following sources: ____________________________________________________________________________________________________________________ | | | | |
| **On a regular base, do you counsel your patients on health and prevention offers?** | Yes  No | | | | |
| **Do you use a digital quality management system?** | Yes, ______________ (please specify)  No | | | | |

## 3. Short questionnaire (preceding qualitative telephone interviews) for GPs participating in GP-centered care

| **Year of birth:** | 19 \|___\|___\| (Please insert year) | | | |
| --- | --- | --- | --- | --- |
| **Sex:** | female | male | | |
| **How many years of professional experience do you have?** | ca. \|___\|___\| years (including further education) | | | |
| **In which year did you take up residence?** | \|___\|___\|___\|___\| (Please insert year) | | | |
| **What is your field of expertise:** | Specialist for general practice | | | |
|  | Internist working as a GP  Other: _______________________________ | | | |
| **Which additional qualifications do you have?** | 1. ________________________________________  2. ________________________________________  3. ________________________________________ | | | |
| **What are the main fields of your practice?** | 1. ________________________________________  2. ________________________________________  3. ________________________________________ | | | |
| **Where is your practice located?** | City center  Urban hinterland (ca. 20 km) | | | Rural area |
| **In which type of practice do you work?** | Individual practice  Shared practice | | | Ambulatory healthcare center  Group practice |
| **How many individual patients do you see per quarter?** | < 500  1001-1500 | | | 500-1000  > 1500 |
| **What is the total full time equivalent of physicians in your practice?** | \|___\|___\|,\|___\| full time physicians | | | |
| **What is the total full time equivalent of physicians’ assistants in your practice?** | \|___\|___\|,\|___\| full time physicians’ assistants | | | |
| **How do you handle documentation in your practice?** | completely digital  completely paper- based  partly digital, partly paper-based | | | |
| **Do you participate in one or more disease management programs?** | No  Yes, heart failure module | | Yes, coronary heart disease  Other | |
| **How many of your patients suffering from coronary heart disease participate in the disease management program for coronary heart disease?** | ca. \|___\|___\|___\| % of patients with coronary heart disease  not applicable | | | |
| **How many of your patients suffering from heart failure participate in the heart failure module?** | ca. \|___\|___\|___\| % of patients with heart failure  not applicable | | | |
| **How many urgent cardiologic emergencies do you have per month that require further diagnostics or a referral to the hospital?** | ca. \|___\|___\|___\| urgent emergencies per month | | | |
| **Since when do you cooperate with the cardiology program** | 20 \|__\|__\| (Please insert year) | | | |
| **How many of your (AOK-) patients participate in the cardiology program?** | ca. \|___\|___\|___\| % | | | |
| **How many times a year do you participate in general practitioner’s quality circles on pharmaceutical therapy?** | ca. \|___\|___\| times a year | | | |
| **How often do you participate in advanced education with a focus on cardiology?** | ca. \|___\|___\| times a year | | | |
| **Do you use a GP-centered care-related software for prescriptions of pharmaceuticals?** | Yes  No | | | |
| **Do you hand out further (evidence-based) information to your patients on managing their illness?** | No  Yes, e.g. from the following sources: __________________________________________________________________________________________________________________ | | | |
| **On a regular base, do you counsel your patients on health and prevention offers?** | Yes  No | | | |
| **Do you use a digital quality management system?** | Yes, ______________ (please specify)  No | | | |
| **Please rate your content with interdisciplinary cooperation in the cardiology program through school grades (1= „very good“, 6 = „insufficient“)** | Grade:_______________ | | | |

## 4. Short questionnaire (preceding qualitative telephone interviews) for GPs not participating in GP-centered care

| **Year of birth:** | 19 \|___\|___\| (Please insert year) | | | |
| --- | --- | --- | --- | --- |
| **Sex:** | female | male | | |
| **How many years of professional experience do you have?** | ca. \|___\|___\| years (including further education) | | | |
| **In which year did you take up residence?** | \|___\|___\|___\|___\| (Please insert year) | | | |
| **What is your field of expertise:** | Specialist for general practice | | | |
|  | Internist working as a GP  Other: _______________________________ | | | |
| **Which additional qualifications do you have?** | 1. ________________________________________  2. ________________________________________  3. ________________________________________ | | | |
| **What are the main fields of your practice?** | 1. ________________________________________  2. ________________________________________  3. ________________________________________ | | | |
| **Where is your practice located?** | City center  Urban hinterland (ca. 20 km) | | | Rural area |
| **In which type of practice do you work?** | Individual practice  Shared practice | | | Ambulatory healthcare center  Group practice |
| **How many individual patients do you see per quarter?** | < 500  1001-1500 | | | 500-1000  > 1500 |
| **What is the total full time equivalent of physicians in your practice?** | \|___\|___\|,\|___\| full time physicians | | | |
| **What is the total full time equivalent of physicians’ assistants in your practice?** | \|___\|___\|,\|___\| full time physicians’ assistants | | | |
| **How do you handle documentation in your practice?** | completely digital  completely paper- based  partly digital, partly paper-based | | | |
| **Do you participate in one or more disease management programs?** | No  Yes, heart failure module | | Yes, coronary heart disease  Other | |
| **How many of your patients suffering from coronary heart disease participate in the disease management program for coronary heart disease?** | ca. \|___\|___\|___\| % of patients with coronary heart disease  not applicable | | | |
| **How many of your patients suffering from heart failure participate in the heart failure module?** | ca. \|___\|___\|___\| % of patients with heart failure  not applicable | | | |
| **How many urgent cardiologic emergencies do you have per month that require further diagnostics or a referral to the hospital?** | ca. \|___\|___\|___\| urgent emergencies per month | | | |
| **How many times a year do you participate in general practitioner’s quality circles on pharmaceutical therapy?** | ca. \|___\|___\| times a year | | | |
| **How often do you participate in advanced education with a focus on cardiology?** | ca. \|___\|___\| times a year | | | |
| **Do you hand out further (evidence-based) information to your patients on managing their illness?** | No  Yes, e.g. from the following sources: __________________________________________________________________________________________________________________ | | | |
| **On a regular base, do you counsel your patients on health and prevention offers?** | Yes  No | | | |
| **Do you use a digital quality management system?** | Yes, ______________ (please specify)  No | | | |

## 5. Questionnaire (quantitative study) for cardiologists participating in the cardiology program

| **Year of birth:** | | 19 \|___\|___\| (Please insert year) | | | | | | | | | | | | | | | | | | | | | | | | | | | | | |
| --- | --- | --- | --- | --- | --- | --- | --- | --- | --- | --- | --- | --- | --- | --- | --- | --- | --- | --- | --- | --- | --- | --- | --- | --- | --- | --- | --- | --- | --- | --- | --- |
| **Sex:** | | female | | | | | | | | | male | | | | | | | | | | | | | | | | | | | | |
| **How many years of professional experience do you have?** | | ca. \|___\|___\| years (including further education) | | | | | | | | | | | | | | | | | | | | | | | | | | | | | |
| **In which year did you take up residence?** | | \|___\|___\|___\|___\| (Please insert year) | | | | | | | | | | | | | | | | | | | | | | | | | | | | | |
| **What is your field of expertise:** | | Internal medicine: | | | | | | | | | without focus  with focus on cardiology | | | | | | | | | | | | | | | | | | | | |
|  |  | Internal medicine and cardiology  Other: _______________________________ | | | | | | | | | | | | | | | | | | | | | | | | | | | | | |
| **Do you perform invasive cardiology?** | | Yes | | | | | | | | | | | | | | No | | | | | | | | | | | | | | | |
| **Do you perform stress echocardiography?** | | Yes | | | | | | | | | | | | | | No | | | | | | | | | | | | | | | |
| **Which additional qualifications do you have?** | | 1. ________________________________________  2. ________________________________________  3. ________________________________________ | | | | | | | | | | | | | | | | | | | | | | | | | | | | | |
| **What are the main fields of your practice?** | | 1. ________________________________________  2. ________________________________________  3. ________________________________________ | | | | | | | | | | | | | | | | | | | | | | | | | | | | | |
| **How often do you participate in advanced education with a focus on cardiology?** | | ca. \|___\|___\| times a year | | | | | | | | | | | | | | | | | | | | | | | | | | | | | |
| **Where is your practice located?** | | City center  Urban hinterland (ca. 20 km) | | | | | | | | | | | | | | | | Rural area | | | | | | | | | | | | | |
| **In which type of practice do you work?** | | Individual practice  Shared practice | | | | | | | | | | | | | | | | Ambulatory healthcare center  Group practice | | | | | | | | | | | | | |
| **Here you see the regions of the state of Baden-Württemberg separated into three groups. Please indicate the group your practice is located in.**  (Please do not indicate the region itself!) | | | | | | | | | | | | | | | | | | | | | | | | | | | | | | | |
| **Group 1:** | | | Region Heilbronn-Franken \| Region Schwarzwald-Baar-Heuberg  Region Ostwürttemberg \| Region Mittlerer Oberrhein/Karlsruhe | | | | | | | | | | | | | | | | | | | | | | | | | | | | |
| **Group 2:** | | | Region Rhein-Neckar \| Region Neckar-Alb  Region Stuttgart \| Region Nordschwarzwald | | | | | | | | | | | | | | | | | | | | | | | | | | | | |
| **Group 3:** | | | Region Hochrhein-Bodensee \| Region Südlicher Oberrhein  Region Bodensee-Oberschwaben \| Region Donau-Iller | | | | | | | | | | | | | | | | | | | | | | | | | | | | |
| **How many individual patients do you see per quarter?** | | < 500  1001-1500 | | | | | | | | | | | | | | | | 500-1000  > 1500 | | | | | | | | | | | | | |
| **Do you use a digital quality management system?** | | Yes, ______________ (please specify)  No | | | | | | | | | | | | | | | | | | | | | | | | | | | | | |
| **What is the total full time equivalent of physicians in your practice?** | | \|___\|___\|,\|___\| full time physicians | | | | | | | | | | | | | | | | | | | | | | | | | | | | | |
| **What is the total full time equivalent of physicians’ assistants in your practice?** | | Up to 3  More than 6, up to 10 | | | | | | | | | | | | More than 3, up to 6  More than 10 | | | | | | | | | | | | | | | | | |
| **Do you employ an EFA in cardiology [type of assistant for medical specialist’s practices]** | | No  Yes, \|___\|___\| EFA | | | | | | | | | | | | | | | | | | | | | | | | | | | | | |
| **How do you handle documentation in your practice?** | | completely digital  completely paper- based  partly digital, partly paper-based | | | | | | | | | | | | | | | | | | | | | | | | | | | | | |
| **How many urgent cardiologic emergencies do you have per month that require further diagnostics or a referral to the hospital?** | | ca. \|___\|___\|___\| urgent emergencies per month | | | | | | | | | | | | | | | | | | | | | | | | | | | | | |
| **Do you offer appointments on each working day?** | | Yes | | | | | | | | No | | | | | | | | | | | | | | | | | | | | | |
| **Do you offer late appointments until 8:00 p.m. or longer at least once a week?** | | Yes, regularly  Yes, but only as an exception  No | | | | | | | | | | | | | | | | | | | | | | | | | | | | | |
| **Since when do you participate in the cardiology program?** | | 20 \|___\|___\| (Please enter year) | | | | | | | | | | | | | | | | | | | | | | | | | | | | | |
| **Please choose a maximum of three aspects especially relevant for your participation in the cardiology program (compared to regular health care).** | | | | | | | | | | | | | | | | | | | | | | | | | | | | | | | |
| Higher reimbursement  More time for patients  Simple accounting  More diagnostic options  Recommendation of the professional association | | | | Alternative to the SHI’s system  Closer cooperation with GPs  Better guideline-adherence of care  Participation/recommendation from colleagues  None | | | | | | | | | | | | | | | | | | | | | | | | | | | |
| Other: | ____________________________________________________________  _____________________________________________________________ | | | | | | | | | | | | | | | | | | | | | | | | | | | | | | |
|  |  |  |  |  |  |  |  |  |  |  |  |  |  |  |  |  |  |  |  |  |  |  |  |  |  |  |  |  |  |  |  |
| **How complex was integration of the cardiology program in your day-to-day business?** | | |  | | |  | | | | | |  | | | | | | | | |  | | | |  | | | |  | | |
|  |  |  | Not complex at all | | | | | | | | |  | | | | | | | | | | | | | Very complex | | | | | | |
| **Did you need to extend your opening hours for participation in the cardiology program?** | | | Yes | | | | | | | | | | | | | | | | | | No | | | | | | | | | | |
| **Your expectations regarding the cardiology program have…** | | | Been fulfilled completely  Been fulfilled largely  Been fulfilled partially  Been fulfilled barely  Not been fulfilled at all  I did not have any expectations | | | | | | | | | | | | | | | | | | | | | | | | | | | | |
| **How many of your (AOK-) patients participate in the cardiology program?** | | | ca. \|___\|___\|___\| % | | | | | | | | | | | | | | | | | | | | | | | | | | | | |
| **Who normally initiates participation of patients in the cardiology program?** | | | Myself  The patient | | | | | | | | | | | | | | | | | | Other medical specialists  The GP | | | | | | | | | | |
| **What is the share of patients in the cardiology program visiting your practice even though they are cardiologically inconspicuous?** | | |  | | | |  | | | | |  | | | | | | | | |  | | | |  | | |  | | | |
|  |  |  | Very small | | | | | | | | |  | | | | | | | | | | | | | Very large | | | | | | |
| **Do you use a cardiology contract-related software for prescriptions of pharmaceuticals?** | | | Yes  No  I don’t know | | | | | | | | | | | | | | | | | | | | | | | | | | | | |
| **The color coding in your contract-related software makes you prescribe discounted pharmaceuticals to participants in the cardiology program...** | | | Less often than in regular care  As often as in regular care  More often than in regular care  I don’t know about the color coding | | | | | | | | | | | | | | | | | | | | | | | | | | | | |
| **Compared to regular care, how much do you profit from the cardiology program?** | | |  | | | |  | | | | | |  | | | | | | | | |  | | | |  | | | |  | |
|  |  |  | To a great extent | | | | | | | | | |  | | | | | | | | | | | | | Not at all | | | | | |
| **Compared to regular care, how much do your participating patients profit from the cardiology program?** | | |  | | | |  | | | | | |  | | | | | | | | |  | | | |  | | | |  | |
|  |  |  | To a great extent | | | | | | | | | |  | | | | | | | | | | | | | Not at all | | | | | |
| **Do you participate in the disease management program for coronary heart disease?** | | | Yes | | | | | | | | | | | | | | | | | No | | | | | | | | | | | |
| **Do you motivate eligible patients participating in the cardiology program to participate in a disease management program?** | | | Yes | | | | | | | | | | | | | | | | | No | | | | | | | | | | | |
| **Did you experience any negative concomitants of the cardiology program?** | | | | | | | | | | | | | | | | | | | | | | | | | | | | | | | |
| No  Yes, the following:  ____________________________________________________________________________  ____________________________________________________________________________  ____________________________________________________________________________  ____________________________________________________________________________  ____________________________________________________________________________ | | | | | | | | | | | | | | | | | | | | | | | | | | | | | | | |
| **Please rate your content with the cardiology program through school grades (1= „very good“, 6 = „insufficient“)** | | | | Grade:_______________ | | | | | | | | | | | | | | | | | | | | | | | | | | | |
| **How long do patients in regular care usually wait for an appointment with you?**  **(Emergencies excluded)** | | | | Up to 2 weeks | | | | | | | | | | | | | More than 2 weeks, up to a month | | | | | | | | | | | | | | |
|  |  |  |  | More than a month, up to 3 months | | | | | | | | | | | | | More than 3 months | | | | | | | | | | | | | | |
| **How long do patients participating in the cardiology program usually wait for an appointment with you?**  **(Emergencies excluded)** | | | | Up to 2 weeks | | | | | | | | | | | | | More than 2 weeks, up to a month | | | | | | | | | | | | | | |
|  |  |  |  | More than a month, up to 3 months | | | | | | | | | | | | | More than 3 months | | | | | | | | | | | | | | |
| **How long does an appointed patient typically wait in your waiting room?**  **(Excluding delays caused by emergencies)** | | | | Up to 30 minutes | | | | | | | | | | | | | | | | | | | | More than 30 minutes, up to 60 minutes | | | | | | | |
|  |  |  |  | More than 60 minutes, up to 90 minutes | | | | | | | | | | | | | | | | | | | | More than 90 minutes | | | | | | | |
| **Do you offer urgent/emergency appointments on the same day?** | | | | Yes | | | | | | | | | | | | | | | | | | | | No | | | | | | | |
| **In your day-to-day work, how much do you adhere to cardiologic guidelines?** | | | |  | | | | |  | | | | | |  | | | | | | | | |  | | |  | | | |  |
|  |  |  |  | Exclusively | | | | | | | | | | |  | | | | | | | | | | | | Not at all | | | | |
| **Which are the guidelines you normally adhere to?**  **(Multiple selections possible)** | | | | DGK-Guidelines  NVL (heart failure)  ACC/AHA-Guidelines | | | | | | | | | | | | | | | | | | | ESC-Guidelines  NVL (coronary heart disease))  Other | | | | | | | | |
| **How much time do you normally spend on the first counseling and information of a patient?** | | | | ca. \|___\|___\|___\| minutes | | | | | | | | | | | | | | | | | | | | | | | | | | | |
| **What are the topics of the first counseling and information of a patient?**  **(Multiple selections possible)** | | | | General information on the disease | | | | | | | | | | | | | | | | | | | | Diet | | | | | | | |
|  |  |  |  | Therapy/medication | | | | | | | | | | | | | | | | | | | | Weight | | | | | | | |
|  |  |  |  | Physical activity/sports | | | | | | | | | | | | | | | | | | | | Addictions (smoking, alcohol…) | | | | | | | |
|  |  |  |  | Other: ____________________________________ | | | | | | | | | | | | | | | | | | | | | | | | | | | |
|  |  |  |  | None of the above | | | | | | | | | | | | | | | | | | | | | | | | | | | |
| **Do you hand out further (evidence-based) information to your patients on managing their illness?** | | | | No  Yes, e.g. from the following sources: _________________________________________________________________________________________________________________________________ | | | | | | | | | | | | | | | | | | | | | | | | | | | |
| **On a regular base, do you counsel your patients on AOK health and prevention offers?** | | | | Yes  No | | | | | | | | | | | | | | | | | | | | | | | | | | | |
| **On a regular base, with how many practice-based GPs do you work together?** | | | | With about \|___\|___\|___\| GPs | | | | | | | | | | | | | | | | | | | | | | | | | | | |
| **How cooperative do you find collaboration with GPs in regular care ?** | | | |  | | | | |  | | | | | |  | | | | | | | | |  | | |  | | | |  |
|  |  |  |  | Not cooperative at all | | | | | | | | | | |  | | | | | | | | | | | | Very cooperative | | | | |
| **How cooperative do you find collaboration with GPs in the cardiology program?** | | | |  | | | | |  | | | | | |  | | | | | | | | |  | | |  | | | |  |
|  |  |  |  | Not cooperative at all | | | | | | | | | | |  | | | | | | | | | | | | Very cooperative | | | | |
| **With patients participating in the cardiology program, how often do you receive the structured “Accompanying letter to the medical specialist” from the GPs including relevant parameters and (presumptive) diagnoses?** | | | |  | | | | |  | | | | | |  | | | | | | | | |  | | |  | | | |  |
|  |  |  |  | Always | | | | | | | | | | |  | | | | | | | | | | | | Never | | | | |
|  |  |  |  | I don’t know about the accompanying letter | | | | | | | | | | | | | | | | | | | | | | | | | | | |
| **Which of the following information on the patient do you usually receive together with the GP’s referral?**  **(Multiple selections possible)** | | | | Relevant (co-) morbidities | | | | | | | | | | | | | | | | | | | Presumptive diagnoses | | | | | | | | |
|  |  |  |  | ICD-Codes | | | | | | | | | | | | | | | | | | | Concrete questions | | | | | | | | |
|  |  |  |  | Laboratory parameters | | | | | | | | | | | | | | | | | | | Medication | | | | | | | | |
|  |  |  |  | Intolerances | | | | | | | | | | | | | | | | | | | DMP-Participations | | | | | | | | |
|  |  |  |  | None of these | | | | | | | | | | | | | | | | | | |  | | | | | | | | |
| **How do you handle transmission of results to the GPs?**  **(Multiple selections possible)** | | | | Via internet  Via fax | | | | | | | | | | | | | | | | | | | Via mail  Other | | | | | | | | |
| **Which of the following information are included in your reports to the GP?**  (Multiple selections possible) | | | | | | | | | | | | | | | | | | | | | | | | | | | | | | | |
| Diagnoses  Instrument-based diagnostics and findings  Summed up evaluation  Pre-medications  Health products | | | | | | | | Laboratory results  ICD-Codes  Anamnesis  Therapy suggestions  Status | | | | | | | | | | | | | | | | | | | | | | | |
| Other: | _________________________________________________________________  _________________________________________________________________ | | | | | | | | | | | | | | | | | | | | | | | | | | | | | | |
|  |  |  |  |  |  |  |  |  |  |  |  |  |  |  |  |  |  |  |  |  |  |  |  |  |  |  |  |  |  |  |  |
| **When are your reports of diagnostic findings sent out to the GP?** | | | | | | | | | | | | | | | | | | | | | | | | | | | | | | | |
| On the same day  Within 5 days | | | | | Within 3 days  Within 6 days or more | | | | | | | | | | | | | | | | | | | | | | | | | | |
| **On which occasions do you call or see respective GPs personally? (Multiple selections possible)** | | | | | | | | | | | | | | | | | | | | | | | | | | | | | | | |
| For regular exchange on patients  For especially complex problems  For maintaining contact | | | | | In case of emergencies/urgent cases  When reports are ambiguous  Never | | | | | | | | | | | | | | | | | | | | | | | | | | |
| **How many times a year do you participate in cardiologically relevant general practitioner’s quality circles on pharmaceutical therapy?** | | | | | ca. \|___\|___\| times a year | | | | | | | | | | | | | | | | | | | | | | | | | | |
| **Besides GPs, with whom do you collaborate regularly?**  **(Multiple selections possible)** | | | | | Fitness centers  Nutritionists | | | | | | | | | | | | | | Sports clubs  Sport clubs for people with heart diseases | | | | | | | | | | | | |
|  |  |  |  |  | Other: ___________________________________  None | | | | | | | | | | | | | | | | | | | | | | | | | | |

## 6. Questionnaire (quantitative study) for cardiologists not participating in the cardiology program

| **Year of birth:** | | | | 19 \|___\|___\| (Please insert year) | | | | | | | | | | | | | |
| --- | --- | --- | --- | --- | --- | --- | --- | --- | --- | --- | --- | --- | --- | --- | --- | --- | --- |
| **Sex:** | | | | female | | | male | | | | | | | | | | |
| **How many years of professional experience do you have?** | | | | ca. \|___\|___\| years (including further education) | | | | | | | | | | | | | |
| **In which year did you take up residence?** | | | | \|___\|___\|___\|___\| (Please insert year) | | | | | | | | | | | | | |
| **What is your field of expertise:** | | | | Internal medicine: | | | without focus  with focus on cardiology | | | | | | | | | | |
|  |  |  |  | Internal medicine and cardiology  Other: _______________________________ | | | | | | | | | | | | | |
| **Do you perform invasive cardiology?** | | | | Yes | | | | | | No | | | | | | | |
| **Do you perform stress echocardiography?** | | | | Yes | | | | | | No | | | | | | | |
| **Which additional qualifications do you have?** | | | | 1. ________________________________________  2. ________________________________________  3. ________________________________________ | | | | | | | | | | | | | |
| **What are the main fields of your practice?** | | | | 1. ________________________________________  2. ________________________________________  3. ________________________________________ | | | | | | | | | | | | | |
| **How often do you participate in advanced education with a focus on cardiology?** | | | | ca. \|___\|___\| times a year | | | | | | | | | | | | | |
| **Where is your practice located?** | | | | City center  Urban hinterland (ca. 20 km) | | | | | | | | | Rural area | | | | |
| **In which type of practice do you work?** | | | | Individual practice  Shared practice | | | | | | | | | Ambulatory healthcare center  Group practice | | | | |
| **Here you see the regions of the state of Baden-Württemberg separated into three groups. Please indicate the group your practice is located in.**  **(Please do not indicate the region itself!)** | | | | | | | | | | | | | | | | | |
| **Group 1:** | | Region Heilbronn-Franken \| Region Schwarzwald-Baar-Heuberg  Region Ostwürttemberg \| Region Mittlerer Oberrhein/Karlsruhe | | | | | | | | | | | | | | | |
| **Group 2:** | | Region Rhein-Neckar \| Region Neckar-Alb  Region Stuttgart \| Region Nordschwarzwald | | | | | | | | | | | | | | | |
| **Group 3:** | | Region Hochrhein-Bodensee \| Region Südlicher Oberrhein  Region Bodensee-Oberschwaben \| Region Donau-Iller | | | | | | | | | | | | | | | |
| **How many individual patients do you see per quarter?** | | | | < 500  1001-1500 | | | | | | | | | | | 500-1000  > 1500 | | |
| **Do you use a digital quality management system?** | | | | Yes, ______________ (please specify)  No | | | | | | | | | | | | | |
| **What is the total full time equivalent of physicians in your practice?** | | | | \|___\|___\|,\|___\| full time physicians | | | | | | | | | | | | | |
| **What is the total full time equivalent of physicians’ assistants in your practice?** | | | | Up to 3  More than 6, up to 10 | | | | | | | | | | | More than 3, up to 6  More than 10 | | |
| **Do you employ an EFA in cardiology [type of assistant for medical specialist’s practices]** | | | | No  Yes, \|___\|___\| EFA | | | | | | | | | | | | | |
| **How do you handle documentation in your practice?** | | | | completely digital  completely paper- based  partly digital, partly paper-based | | | | | | | | | | | | | |
| **How many urgent cardiologic emergencies do you have per month that require further diagnostics or a referral to the hospital?** | | | | ca. \|___\|___\|___\| urgent emergencies per month | | | | | | | | | | | | | |
| **Do you offer appointments on each working day?** | | | | Yes | | | | | No | | | | | | | | |
| **Do you offer late appointments until 8:00 p.m. or longer at least once a week?** | | | | Yes, regularly  Yes, but only as an exception  No | | | | | | | | | | | | | |
| **What were your reasons to decide against participation in the cardiology program (Multiple selections possible)** | | | | | | | | | | | | | | | | | |
| Costs | | | | Administrative efforts | | | | | | | | | | | | | |
| Software conversions | | | | Professional political aspects | | | | | | | | | | | | | |
| Fear for my professional autonomy | | | | Fear for continuity of the SHI system | | | | | | | | | | | | | |
| Lack of eligible patients | | | | I cannot/I do not want to fulfill all of the contract’s criteria | | | | | | | | | | | | | |
| Regional lack of GPs participating in GP-centered care | | | | Colleagues advised me against the program | | | | | | | | | | | | | |
| I don’t know the cardiology program | | | |  | | | | | | | | | | | | | |
| Other: | _______________________________________________________________  _______________________________________________________________ | | | | | | | | | | | | | | | | |
|  |  |  |  |  |  |  |  |  |  |  |  |  |  |  |  |  |  |
| **How long do patients usually wait for an appointment with you?**  **(Emergencies excluded)** | | | | Up to 2 weeks | | | | | | | | | | | More than 2 weeks, up to a month | | |
|  |  |  |  | More than a month, up to 3 months | | | | | | | | | | | More than 3 months | | |
| **How long does an appointed patient typically wait in your waiting room?**  **(Excluding delays caused by emergencies)** | | | | Up to 30 minutes | | | | | | | | | | | More than 30 minutes, up to 60 minutes | | |
|  |  |  |  | More than 60 minutes, up to 90 minutes | | | | | | | | | | | More than 90 minutes | | |
| **What is the share of patients in the cardiology program visiting your practice even though they are cardiologically inconspicuous?** | | | |  | |  | |  | | | | | | |  |  |  |
|  |  |  |  | Very small | | | |  | | | | | | | | Very large | |
| **Do you participate in the disease management program for coronary heart disease?** | | | | Yes | | | | | | | | | | | No | | |
| **Do you motivate eligible patients participating in the cardiology program to participate in a disease management program?** | | | | Yes | | | | | | | | | | | No | | |
| **Do you offer urgent/emergency appointments on the same day?** | | | | Yes | | | | | | | | | | | No | | |
| **In your day-to-day work, how much do you adhere to cardiologic guidelines?** | | | |  | |  | |  | | | | | | |  |  |  |
|  |  |  |  | Exclusively | | | |  | | | | | | | | Not at all | |
| **Which are the guidelines you normally adhere to?**  **(Multiple selections possible)** | | | | DGK-Guidelines  NVL (heart failure)  ACC/AHA-Guidelines | | | | | | | | | ESC-Guidelines  NVL (coronary heart disease))  Other | | | | |
| **How much time do you normally spend on the first counseling and information of a patient?** | | | | ca. \|___\|___\|___\| minutes | | | | | | | | | | | | | |
| **What are the topics of the first counseling and information of a patient?**  **(Multiple selections possible)** | | | | General information on the disease | | | | | | | | Diet | | | | | |
|  |  |  |  | Therapy/medication | | | | | | | | Weight | | | | | |
|  |  |  |  | Physical activity/sports | | | | | | | | Addictions (smoking, alcohol…) | | | | | |
|  |  |  |  | Other: ____________________________________ | | | | | | | | | | | | | |
|  |  |  |  | None of the above | | | | | | | | | | | | | |
| **Do you hand out further (evidence-based) information to your patients on managing their illness?** | | | | No  Yes, e.g. from the following sources: ________________________________________________________________________________________________________ | | | | | | | | | | | | | |
| **On a regular base, do you counsel your patients on health insurer’s health and prevention offers?** | | | | Yes  No | | | | | | | | | | | | | |
| **On a regular base, with how many practice-based GPs do you work together?** | | | | With about \|___\|___\|___\| GPs | | | | | | | | | | | | | |
| **How cooperative do you usually find collaboration with GPs?** | | | |  | |  | |  | | | | | | |  |  |  |
|  |  |  |  | Not cooperative at all | | | |  | | | | | | | | Very cooperative | |
| **Which of the following information on the patient do you usually receive together with the GP’s referral?**  **(Multiple selections possible)** | | | Relevant (co-) morbidities | | | | | | | | | | | Presumptive diagnoses | | | |
|  |  |  | ICD-Codes | | | | | | | | | | | Concrete questions | | | |
|  |  |  | Laboratory parameters | | | | | | | | | | | Medication | | | |
|  |  |  | Intolerances | | | | | | | | | | | DMP-Participations | | | |
|  |  |  | None of these | | | | | | | | | | |  | | | |
| **How do you handle transmission of results to the GPs?**  **(Multiple selections possible)** | | | Via internet  Via fax | | | | | | | | Via mail  Other | | | | | | |
| **Which of the following information are included in your reports to the GP?**  **(Multiple selections possible)** | | | | | | | | | | | | | | | | | |
| Diagnoses  Instrument-based diagnostics and findings  Summed up evaluation  Pre-medications  Health products | | | Laboratory results  ICD-Codes  Anamnesis  Therapy suggestions  Status | | | | | | | | | | | | | | |
| Other: | __________________________________ | | | | | | | | | | | | | | | | |
|  | __________________________________ | | | | | | | | | | | | | | | | |
| **When are your reports of diagnostic findings sent out to the GP?** | | | | | | | | | | | | | | | | | |
| On the same day  Within 5 days | | | | | Within 3 days  Within 6 days or more | | | | | | | | | | | | |
| **On which occasions do you call or see respective GPs personally? (Multiple selections possible)** | | | | | | | | | | | | | | | | | |
| For regular exchange on patients  For especially complex problems  For maintaining contact | | | | | In case of emergencies/urgent cases  When reports are ambiguous  Never | | | | | | | | | | | | |
| **How many times a year do you participate in cardiologically relevant general practitioner’s quality circles on pharmaceutical therapy?** | | | ca. \|___\|___\| times a year | | | | | | | | | | | | | | |
| **Besides GPs, with whom do you collaborate regularly?**  **(Multiple selections possible)** | | | Fitness centers  Nutritionists | | | | | | | | Sports clubs  Sport clubs for people with heart diseases | | | | | | |
|  |  |  | Other: ___________________________________  None | | | | | | | | | | | | | | |

## 7. Questionnaire (quantitative study) for GPs participating in the GP-centered care

| **Year of birth:** | 19 \|___\|___\| (Please insert year) | | | | | | | | | | | | | | | | | |
| --- | --- | --- | --- | --- | --- | --- | --- | --- | --- | --- | --- | --- | --- | --- | --- | --- | --- | --- |
| **Sex:** | female | | | | | male | | | | | | | | | | | | |
| **How many years of professional experience do you have?** | ca. \|___\|___\| years (including further education) | | | | | | | | | | | | | | | | | |
| **In which year did you take up residence?** | \|___\|___\|___\|___\| (Please insert year) | | | | | | | | | | | | | | | | | |
| **What is your field of expertise:** | Specialist for general practice | | | | | | | | | | | | | | | | | |
|  | Internal specialist working as a GP  Other: _______________________________ | | | | | | | | | | | | | | | | | |
| **Which additional qualifications do you have?** | 1. ________________________________________  2. ________________________________________  3. ________________________________________ | | | | | | | | | | | | | | | | | |
| **What are the main fields of your practice?** | 1. ________________________________________  2. ________________________________________  3. ________________________________________ | | | | | | | | | | | | | | | | | |
| **How often do you participate in advanced education with a focus on cardiology?** | ca. \|___\|___\| times a year | | | | | | | | | | | | | | | | | |
| **Where is your practice located?** | City center  Urban hinterland (ca. 20 km) | | | | | | | | | Rural area | | | | | | | | |
| **In which type of practice do you work?** | Individual practice  Shared practice | | | | | | | | | Ambulatory healthcare center  Group practice | | | | | | | | |
| **Here you see the regions of the state of Baden-Württemberg separated into three groups. Please indicate the group your practice is located in.**  **(Please do not indicate the region itself!)** | | | | | | | | | | | | | | | | | | |
| **Group 1:** | | | Region Heilbronn-Franken \| Region Schwarzwald-Baar-Heuberg  Region Ostwürttemberg \| Region Mittlerer Oberrhein/Karlsruhe | | | | | | | | | | | | | | | |
| **Group 2:** | | | Region Rhein-Neckar \| Region Neckar-Alb  Region Stuttgart \| Region Nordschwarzwald | | | | | | | | | | | | | | | |
| **Group 3:** | | | Region Hochrhein-Bodensee \| Region Südlicher Oberrhein  Region Bodensee-Oberschwaben \| Region Donau-Iller | | | | | | | | | | | | | | | |
| **How many individual patients do you see per quarter?** | < 500  1001-1500 | | | | | | | | | 500-1000  > 1500 | | | | | | | | |
| **Do you use a digital quality management system?** | Yes, ______________ (please specify)  No | | | | | | | | | | | | | | | | | |
| **What is the total full time equivalent of physicians in your practice?** | \|___\|___\|,\|___\| full time physicians | | | | | | | | | | | | | | | | | |
| **What is the total full time equivalent of physicians’ assistants in your practice?** | Up to 3  More than 6, up to 10 | | | | | | | More than 3, up to 6  More than 10 | | | | | | | | | | |
| **How do you handle documentation in your practice?** | completely digital  completely paper- based  partly digital, partly paper-based | | | | | | | | | | | | | | | | | |
| **How many urgent cardiologic emergencies do you have per month that require further diagnostics or a referral to the hospital?** | ca. \|___\|___\|___\| urgent emergencies per month | | | | | | | | | | | | | | | | | |
| **Since when do you cooperate with the cardiology program?** | 20 \|___\|___\|___\| (please insert year) | | | | | | | | | | | | | | | | | |
| **Was did you expect regarding the cardiology program compared to regular care? (Multiple selections possible)** | | | | | | | | | | | | | | | | | | |
| Faster appointments for my participating patients with a medical specialist | | A closer collaboration with medical specialists | | | | | | | | | | | | | | | | |
| More time for my participating patients at the medical specialist | | A more guideline-adhering care for patients | | | | | | | | | | | | | | | | |
| More diagnostic options at the medical specialist | | Nothing | | | | | | | | | | | | | | | | |
| Other: | | _________________________________________________  _________________________________________________ | | | | | | | | | | | | | | | | |
|  | |  |  |  |  |  |  |  |  |  |  |  |  |  |  |  |  |  |
| **Your expectations regarding the cardiology program have…** | | | Been fulfilled completely  Been fulfilled largely  Been fulfilled partially  Been fulfilled barely  Not been fulfilled at all  I did not have any expectations | | | | | | | | | | | | | | | |
| **How many of your (AOK-) patients participate in the cardiology program?** | | | ca. \|___\|___\|___\| % | | | | | | | | | | | | | | | |
| **Who normally initiates participation of patients in the cardiology program?** | | | Myself  The patient | | | | | | Other medical specialists  The GP | | | | | | | | | |
| **Compared to regular care, how much do you profit from the cardiology program?** | | |  | |  | |  | | | |  | | | |  | |  | |
|  |  |  | To a great extent | | | |  | | | | | | | | Not at all | | | |
| **Compared to regular care, how much do your participating patients profit from the cardiology program?** | | |  | |  | |  | | | |  | | | |  | |  | |
|  |  |  | To a great extent | | | |  | | | | | | | | Not at all | | | |
| **Did you experience any negative concomitants of the cardiology program?** | | | | | | | | | | | | | | | | | | |
| No  Yes, the following:  ____________________________________________________________________________  ____________________________________________________________________________  ____________________________________________________________________________  ____________________________________________________________________________  ____________________________________________________________________________ | | | | | | | | | | | | | | | | | | |
| **Please rate cooperation with the cardiology program through school grades (1= „very good“, 6 = „insufficient“)** | | | Grade:_______________ | | | | | | | | | | | | | | | |
| **How long do patients usually wait for an appointment with you?**  **(Emergencies excluded)** | | | Up to 2 days | | | | | | | | | More than 2 days, up to 5 days | | | | | | |
|  |  |  | More than 5 days, up to 10 days | | | | | | | | | More than 10 days | | | | | | |
| **Do you offer urgent/emergency appointments on the same day?** | | | Yes | | | | | | | | | No | | | | | | |
| **In your day-to-day work, how much do you adhere to cardiologic guidelines?** | | |  | |  | |  | | | | |  | | | |  | |  |
|  |  |  | Exclusively | | | |  | | | | | | | | | Not at all | | |
| **Which are the guidelines you normally adhere to?**  **(Multiple selections possible)** | | | DGK-Guidelines  NVL (heart failure)  ACC/AHA-Guidelines  Other | | | | | | | | | ESC-Guidelines  NVL (coronary heart disease))  DEGAM (chest pain)  None | | | | | | |
| **Do you hand out further (evidence-based) information to your patients on managing their illness?** | | No  Yes, e.g. from the following sources: ____________________________________________________________________________________________________________ | | | | | | | | | | | | | | | | |
| **On a regular base, do you counsel your patients on health insurer’s health and prevention offers?** | | Yes  No | | | | | | | | | | | | | | | | |
| **On a regular base, with how many practice-based cardiologists do you work together?** | | With about \|___\|___\|___\| cardiologists | | | | | | | | | | | | | | | | |
| **How cooperative do you usually find collaboration with cardiologists in regular care?** | |  | |  | |  | | | | |  | | | |  | |  | |
|  |  | Not cooperative at all | | | |  | | | | | | | | | Very cooperative | | | |
| **How cooperative do you usually find collaboration with cardiologists in the cardiology program/GP-centered care?** | |  | |  | |  | | | | |  | | | |  | |  | |
|  |  | Not cooperative at all | | | |  | | | | | | | | | Very cooperative | | | |
| **With patients participating in the cardiology program, how often do you send the structured “Accompanying letter to the medical specialist” including relevant parameters and (presumptive) diagnoses?** | |  | |  | |  | | | | |  | | | |  | |  | |
|  |  | Always | | | |  | | | | | | | | | Never | | | |
|  |  | I don’t know about the accompanying letter | | | | | | | | | | | | | | | | |
| **Which of the following information on the patient do you usually send together with your referral to the cardiologist?**  **(Multiple selections possible)** | | Relevant (co-) morbidities | | | | | | | | | | | | Presumptive diagnoses | | | | |
|  |  | ICD-Codes | | | | | | | | | | | | Concrete questions | | | | |
|  |  | Laboratory parameters | | | | | | | | | | | | Medication | | | | |
|  |  | Intolerances | | | | | | | | | | | | DMP-Participations | | | | |
|  |  | None of these | | | | | | | | | | | |  | | | | |
| **How do you handle transmission of results to the GPs?**  **(Multiple selections possible)** | | Via internet  Via fax | | | | | | | | | | | | Via mail  Other | | | | |
| **Which of the following information are included in the cardiologists reports to you ?**  **(Multiple selections possible)** | | | | | | | | | | | | | | | | | | |
| Diagnoses  Instrument-based diagnostics and findings  Summed up evaluation  Pre-medications  Health products | | Laboratory results  ICD-Codes  Anamnesis  Therapy suggestions  Status | | | | | | | | | | | | | | | | |
| Other: | | __________________________________________________  __________________________________________________ | | | | | | | | | | | | | | | | |
|  | |  |  |  |  |  |  |  |  |  |  |  |  |  |  |  |  |  |
| **After your patient’s visit, when do usually you receive the cardiologists reports of diagnostic findings?** | | On the same day  Within 5 days | | | | | | | | | | | Within 3 days  Within 6 days or more | | | | | |
| **Compared to regular care, exchange of diagnostic findings with cardiologists in the cardiology program is…** | | Much better  A little better  Not different  A little worse  Much worse | | | | | | | | | | | | | | | | |
| **In urgent cases, do you get appointments with the cardiologist on the same day for your patients?** | | Yes, regularly  Yes, but only for patients in the cardiology program  Rarely  No | | | | | | | | | | | | | | | | |
| **How many times a year do you participate in general practitioner’s quality circles on pharmaceutical therapy?** | | ca. \|___\|___\| times a year | | | | | | | | | | | | | | | | |
| **Besides cardiologists, with whom do you collaborate regularly?**  **(Multiple selections possible)** | | Fitness centers  Nutritionists | | | | | | | | | | | Sports clubs  Sport clubs for people with heart diseases | | | | | |
|  |  | Other: ___________________________________  None | | | | | | | | | | | | | | | | |

*All questionnaires were translated from German to English by the authors.
