# Supplementary material for: Implementing coordinated ambulatory cardiology care in southern Germany: a mixed-methods study
Source: BMC Health Serv Res. 2019 Dec 19;19:976. doi: 10.1186/s12913-019-4832-4 (PMC6921457; doi:10.1186/s12913-019-4832-4)
Supplement: Supplementary file 2 — Additional file 2. Questions from the qualitative interview guides on implementation of the cardiology program. [file 12913_2019_4832_MOESM2_ESM.docx]

# Additional file 2: Questions from the qualitative interview guides on implementation of the cardiology program*

## Questions for cardiologists participating in the cardiology program

Patient care

1. Did the cardiology program initiate some kind of rethinking within your practice?
   - 1. In your opinion, what has changed in your day-to-day business?
2. Did patient care change because of the program?
   1. Can you tell us what exactly changed?
3. How important are guidelines in your day-to-day business?
   1. When do you deviate (on purpose) from guidelines?
4. What do you think about the contract-related software for the prescription of pharmaceuticals?
   1. Did it change your prescription habits?

Contextual factors

1. Do you think there are enough medical specialists for cardiovascular diseases in your area?
2. Did you experience any factors outside of the cardiology program that influenced its implementation?

Cooperation

1. Please describe your cooperation with GPs
   - 1. Did cooperation with GPs change because of your participation in the cardiology program?
        1. Can you describe what exactly changed?
           1. With how many GPs do you collaborate?
           2. How do you exchange information with GPs?
           3. What do you think about documentation and exchange of diagnostic findings via the contract-related software?

When do you transmit diagnostic findings to the GP?

Administrative processes/practice structures

1. Did you change any processes/routines in your practice because of the cardiology program?
   1. What exactly did you change?
      1. Did making appointments change?
      2. When do patients (in the cardiology program) get an appointment?
      3. Do you offer early or late appointments for working patients?
      4. How much time do patients usually spend in your waiting room?
      5. Did you change emergency management?
2. Do you think your practice is well-staffed??

## Questions for cardiologists not participating in the cardiology program

Contextual factors

1. Do you think there are enough medical specialists for cardiovascular diseases in your area?
2. Did you experience any factors outside of the cardiology program that influenced its implementation for participating physicians?

## Questions for GPs participating in GP-centered care

Contextual factors

1. Do you think there are enough medical specialists for cardiovascular diseases in your area?
2. Did you experience any factors outside of the cardiology program that influenced its implementation?

Cooperation

1. Please describe your cooperation with cardiologists
   - 1. Did cooperation with cardiologists change because of your participation in the cardiology program?
        1. Can you describe what exactly changed?
           1. With how many cardiologists do you collaborate?
           2. How do you exchange information with cardiologists?
           3. What do you think about documentation and exchange of diagnostic findings via the contract-related software?

When do you transmit diagnostic findings to the cardiologist?

## Questions for GPs not participating in GP-centered care

Contextual factors

1. Do you think there are enough medical specialists for cardiovascular diseases in your area?
2. Did you experience any factors outside of the cardiology program that influenced its implementation for participating physicians?

*All questions were translated from German to English by the authors.
