# Supplementary material for: Implementing coordinated ambulatory cardiology care in southern Germany: a mixed-methods study
Source: BMC Health Serv Res. 2019 Dec 19;19:976. doi: 10.1186/s12913-019-4832-4 (PMC6921457; doi:10.1186/s12913-019-4832-4)
Supplement: Supplementary file 3 — Additional file 3. Themes/categories and representative quotes from the qualitative data analysis relevant to the implementation of the cardiology program. [file 12913_2019_4832_MOESM3_ESM.docx]

# Additional file 3: Themes/categories and representative quotes from the qualitative data analysis relevant to the implementation of the cardiology program*

| **Main theme: Implementation of the cardiology program** | | |
| --- | --- | --- |
| **Subsections** | **Categories in qualitative analysis** | **Representative quotes** |
| **Access to cardiology care** |  |  |
|  | Appointments | - *“You have this official duty to offer an appointment within two weeks and because of that we block appointments.”* - *“With patients [participating in the cardiology program] we pay attention so they get faster appointments, because we know it’s written in the contract, but it’s…if I listen to my assistants on the phone, they never ask: ‘Are you [participating in the cardiology contract]? Okay, then we’ll need to get you an early appointment.’, but if they [the patients] accept an appointment, it’s alright and if not, we’d need to see what to do with it.”* |
|  | Consultation hours | - *„My working hours are from 7:15 a.m. […] to 7:00 p.m. […]. Nonstop, without lunch break. Well, not every day. Today for example I finish at noon. But two times a week there are these times and two times a week a little shorter, towards evening. But always the same in the morning, 7:15 a.m.“* - *“So, we have…four afternoons until 6:00 p.m. and sometimes until 7:00 p.m. […] But early, well, we start at 8:00 a.m., there are no appointments before 8:00 a.m. because we think ‘Well, sometime it will also work for someone in the evening’. But at 7:00 p.m. I had some bad experiences, already at the hospital. You have a lot of drop-outs then.”* |
| **Medical care** |  |  |
|  | Guideline-adherence | - *“Our, my principle, guideline of my practice is: guideline-compatible and individual treatment. […] We adhere to guidelines, but still we do it tailored to the individual patient, of course.”* - *“Deviation from guidelines happens when patients do not tolerate pharmaceuticals in a certain dosage or if they refuse to take them. Then you’re forced to deviate. Otherwise, normally not – but I don’t look them [the guidelines] up, but, well, you just internalize them.”* |
|  | Prescriptions | - *“Compared to the beginning, I became more cost-conscious because the software suggests respective pharmaceuticals preferred in the cardiology program through green, red, blue or white color and I put that into practice because the cardiology program demands that you play by the rules of the game and don’t prescribe the most expensive pharmaceuticals. But sometimes there is no other possibility and I need to stretch this rule or break it -well, break it- then there simply is no other way and then a drug is prescribed which isn’t favored, but this is the exception […].”* - *“Of course, we stick to what is discounted mainly, let’s say, if there are no good reasons to decide against it”.* |
| **Cooperation between specialists and GPs** |  |  |
|  | Referrals/exchange of diagnostic findings | - *“It differs a lot. […] you could say there are idiots who send somebody over because of a supposedly striking electrocardiogram and they don’t even send the electrocardiogram, yes. Then you have no idea what to look for, what to aim at. The patients come in and say: ‘Well, the general practitioner shook his head over seeing the electrocardiogram: ‘A specialist needs to see this.’.’ and things like that. From such nonsense to very well prepared patients. But this somehow also depends on the way general practitioners manage their practice.* - *“There are these structured letters, but I don’t use them. So, no, not really. […] Because my letters are relatively structured anyway, with coded diagnoses and I don’t see a reason to…[…] it already writes…echocardiography results are already contained and diagnoses are contained, so no.”* - *“There is this beautiful accompanying letter ought to be sent [by the GP]. We also use this information, but either it arrives blank or nothing arrives at all […]. If patients don’t pick their data up, you have no option, you don’t know yesterday’s parameters, you don’t know how many pharmaceuticals patients take. You can either get on the phone and call everywhere or you need to make a new appointment. That’s really bad.”* |
|  | Keeping in touch with each other | - *“Well, personal contact only if there is something urgent. They [GPs] don’t call on a regular base, but in most cases they give a referral to their patient […]. Urgent cases are via phone, sure. That’s not a problem. Return of information is written, or if it’s something critical so you have to talk or something like that. But this…one hundred percent a letter in each case, yes. And the flow of information from the GP’s practice towards us is pretty diverse, yes. From basically nothing where you at best can ask for something actively up to prepared visits where they have necessary data in advance. It’s very, very diverse.”* - *“There is also a regular’s table. But that’s rather private. […] A local regular’s table. By physicians. […] Twice a year.* - *“It’s very heterogeneous of course. There are colleagues which we call nearly every day and other colleagues we only know the names of, others we know from advanced training. So this is very, very diverse.”* - *“Yes, there is personal contact. We organize quality circles together with the colleagues from GP-centered care. And we organize a lot of in-house advanced training for general practitioners.”* |
| **Contextual factors** |  |  |
|  | Regional aspects | - *“We are in an area, the insurer’s heartland […] and therefore here the cardiology program had been developed, publicized and somehow tested. So, more or less, we have been the pioneering region. Everyone is involved […].”* - *“[…] a lot of people in our region already participated in GP-centered care, because our district medical officer pushed it heavily. Therefore, it was clear that a lot of patients were eligible [for the cardiology program], which hasn’t been the case in other administrative districts.”* - *“In our administrative district, our town, everyone participates [in the cardiology program].”* - *“[…] I have mixed feelings. In the beginning it was really fair, I favored the critically ill, they got their appointments faster. Now, in our state, in this town there are so many participants in this medical specialist’s program that…well, this advantage is completely weakened.”* - *“And, of course, there is no hospital anymore in town, we used to have one. That’s a big issue, because then a lot of emergencies which used to be dealt with by the hospital inevitably appear in this practice.”* - *“I live in the countryside, where you talk to each other. […] It’s not like it is in the city.”* |
|  | Practice’s staff resources/capabilities | - *“I am lucky to have a team which is very competent, very polite, very efficient. […] two out of the three are really good. And they act accordingly, so we get positive feedback for them not letting people alone […] they learned it…we need to solve a problem […].”* - *“Currently, personnel recruitment is the problem. […] That’s really becoming a problem, those with qualifications are getting thinned out more and more. We had four or five trainees and the supply is getting thinned out more and more – wo cares about becoming a physician’s assistant, yes? It’s becoming more and more of a problem, the search as well. Hospitals are also exhausting the market, as they try to compensate their shortage of nursing staff through physician’s assistants. […] It’s really difficult, I experienced it myself because of a pregnancy a year ago and it was extremely difficult until we had a substitute employee”.* - *“Currently we are well-equipped, staff-wise. Regarding physician’s assistants, definitely. We are rather overrepresented currently, as we…well, we currently have four trainees and just accepted another trainee who quit some time ago and wanted to return […]. Of course, this can already change the day after tomorrow, because sometimes two [physician’s assistants] resign at the same time and then you’re…sometimes it’s difficult to staff a vacant position, it can fluctuate a lot.”* |
|  | Information technology | - *“[…] I need to know the accounting numbers I have. Every patient needs to be retrieved through the software, yes. Furthermore, you can ask them in person, not a bad thing. Furthermore, it’s written on the referral normally. So you have a lot of possibilities. It’s only bad, if you need to, and this is an area of concern, you basically need to check everything manually and enter everything manually. And if you don’t do it, the assistant needs to do it. And if she doesn’t pay attention and the doctor doesn’t pay attention and nobody pays attention, then you have an error in accounting. You need to set the software accordingly, if [every time] it’s about the cardiology program. If you don’t do it, you can [accidentally] enter numbers connected to the accountings for [patients in] statutory health insurance – then the drama starts.”* - *“Recently, for example, there was an update and now that dumb device connecting us to GP-centered care doesn’t work again – and accounting deadline is 10 days away and our assistants are already nervous about our IT-service provider -and that’s the way it seems- not going to make it [in time] so we can’t transfer the accounting for patients in the cardiology program again. Such things are compromising. And then, of course, you ask yourself if it’s worth it.”* |
| *All themes and quotes were translated from German to English by the authors. | | |
